# Supplementary material for: An Antarctic ecosystem value index to quantify ecological value across trophic levels and over time
Source: Nat Commun. 2026 Feb 11;17:3203. doi: 10.1038/s41467-026-69011-0 (PMC13056982; doi:10.1038/s41467-026-69011-0)
Supplement: Supplementary file 1 — Supplementary Information [file 41467_2026_69011_MOESM1_ESM.pdf]

Supplementary Material: An Antarctic ecosystem value index to quantify ecological value across trophic levels and over time

Alice K. DuVivier[1*], Kristen M. Krumhardt[1], Laura L. Landrum[1], Zephyr Sylvester[2], Bilgecan Şen[3,4], Sara Labrousse[5], Christian Che-Castaldo[6], Alice Eparvier[3,7], Marika M. Holland[1], Michelle A. LaRue[8], Cara Nissen[9,10], Michael N. Levy[1], Stephanie Jenouvrier[5†], Cassandra Brooks[2,9†]

[1]National Center for Atmospheric Research, 1850 Table Mesa Drive, Boulder, 80305, Colorado, USA.
[2]Environmental Studies Department, University of Colorado at Boulder, Boulder, Colorado, USA.
[3]Woods Hole Oceanographic Institution, Woods Hole, Massachusetts, USA.
[4]Center for Environmental Science, University of Maryland, Cambridge, Maryland, USA.
[5]LOCEAN-IPSL, Sorbonne Université, Paris, France.
[6]U.S. Geological Survey, Wisconsin Cooperative Wildlife Research Unit, Department of Forest and Wildlife Ecology, University of Wisconsin-Madison, Madison, Wisconsin, USA.
[7]Master de Biologie, École Normale Supérieure de Lyon, Université Claude Bernard Lyon 1, Université de Lyon, Lyon, 69007, France.
[8]School of Earth and Environment, Te Whare Wnanga o Waitaha - University of Canterbury, Christchurch,New Zealand.
[9]Institute of Arctic and Alpine Research, University of Colorado at Boulder, Boulder, Colorado, USA.
[10]Department of Freshwater and Marine Ecology, Institute for Biodiversity and Ecosystem Dynamics, University of Amsterdam, Amsterdam, The Netherlands.

*Corresponding author(s). E-mail(s): duvivier@ucar.edu;

†These authors contributed equally to conceptualizing this work.

1 Supplementary Tables

Supplementary Table 1 AEV Index input data source

Antarctic Ecosystem Value (AEV) Index Inputs		
Metric	Historical Reconstruction	Earth System Model
NPP[1]	FOSI[6] [1]	CESM2-LE[7] [2]
KGP[2]	FOSI[6] [1] + Empirical [3]	CESM2-LE[7] [2] + Empirical [3]
DFP[3]	FOSI[6] [1] + FEISTY[8] [4]	CESM2-LE[7] [2] + FEISTY[8] [4]
EPP[4]	Satellite estimate [5]	CESM2-LE[7] [2] + demographic model [6]
APP[5]	Satellite estimate [7]	CESM2-LE[7] [2] + demographic model [8]

[1]Net primary productivity (NPP)

[2]Krill growth potential (KGP)

[3]Demersal fish biomass potential (DFP)

[4]Emperor penguin (*Aptenodytes forsteri*) population (EPP)

[5]Adélie penguin (*Pygoscelis adeliae*) population (APP)

[6]Forced ocean-sea ice (FOSI) model

[7]Community Earth System Model version 2 Large Ensemble (CESM2-LE)

[8]Fisheries Size and Functional Type (FEISTY) model

2 Supplementary Figures

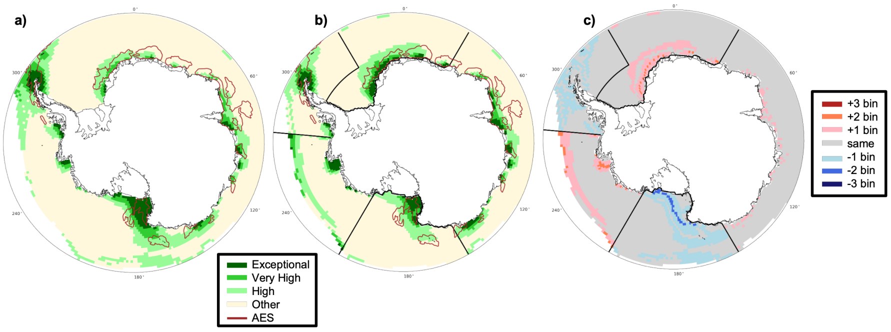

Supplementary Figure 1 Historical reconstruction Antarctic Ecosystem Value (AEV) Index bins scaled a) hemispherically and b) regionally (these panels are identical to those shown on Figure 1a,b in Main Text). Magenta contours on panels a) and b) correspond to Areas of Ecological Significance (AES) identified by 9. c) Difference (regional - hemispheric) in bin classification where reds (blues) indicate that the regional bin classification is more (less) valuable than the hemispheric classification. Coastlines were provided by the US National Ice Center [10].

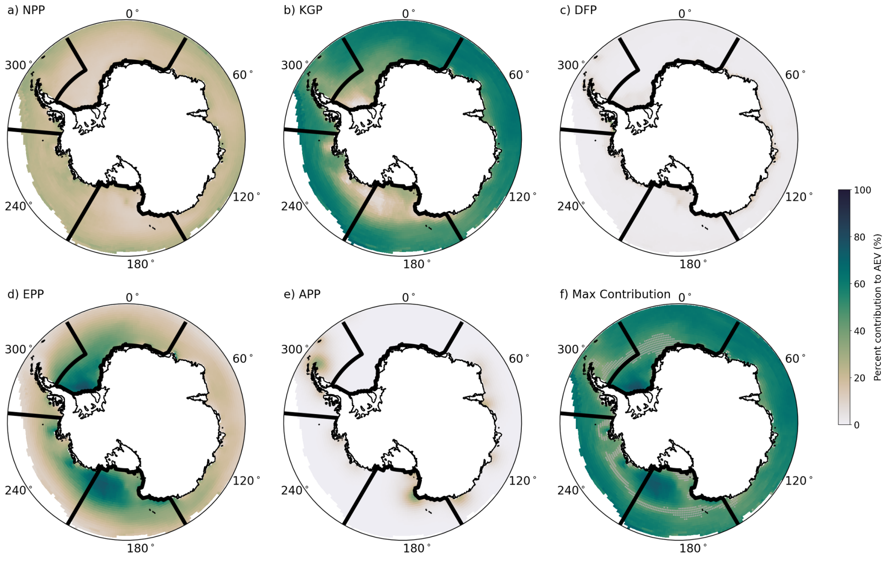

Supplementary Figure 2 Historical reconstruction Antarctic Ecosystem Value (AEV) Index input contributions. The percent contribution at each grid point is provided for a) net primary productivity (NPP), b) krill growth potential (KGP), c) demersal fish biomass potential (DFP), d) Emperor penguin (*Aptenodytes forsteri*) population (EPP), and e) Adélie penguin (*Pygoscelis adeliae*) population (APP). Values range from 0-100 and sum to 100 over all input layers. The maximum input contribution at each point is shown in panel f) where grey stippling indicates points where the maximum input contribution is less than 10% different from the next highest input layer percent contribution. Coastlines were provided by the US National Ice Center [10].

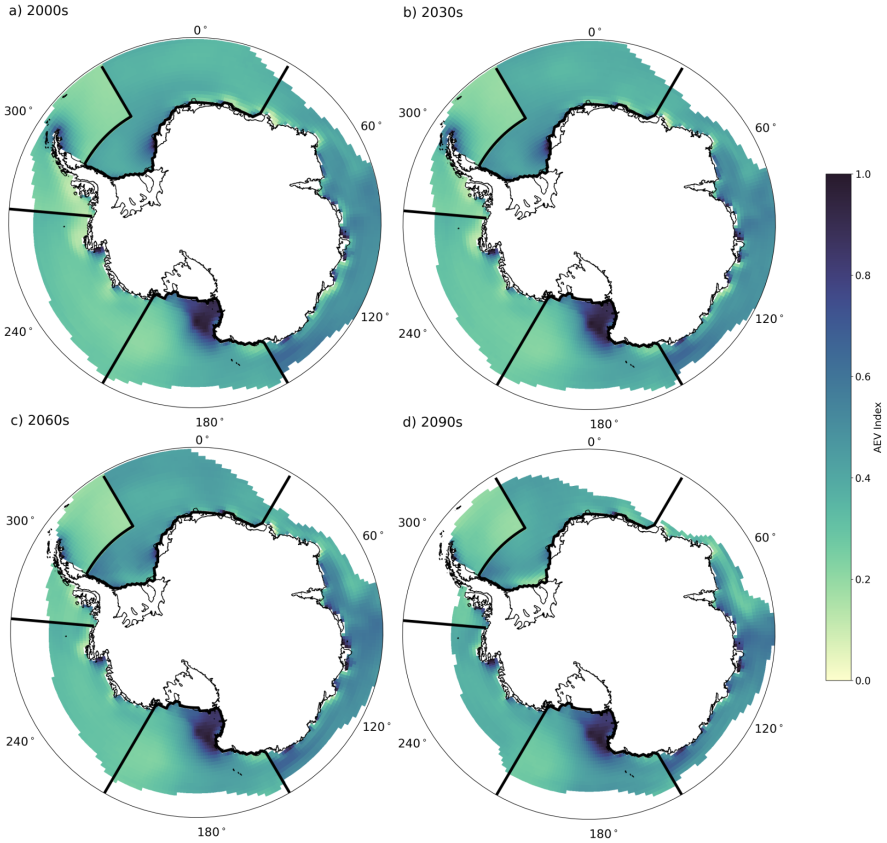

Supplementary Figure 3 Regional Antarctic Ecosystem Value (AEV) Index values for the a) 2000s, b) 2030s, c) 2060s, and d) 2090s. AEV Index values should only be directly compared within a single region and decade, not between decades or regions. Note that because the AEV Index is calculated only at grid points within the seasonal sea ice zone, the spatial extent decreases in all decades compared to the 2000s. Coastlines were provided by the US National Ice Center [10].

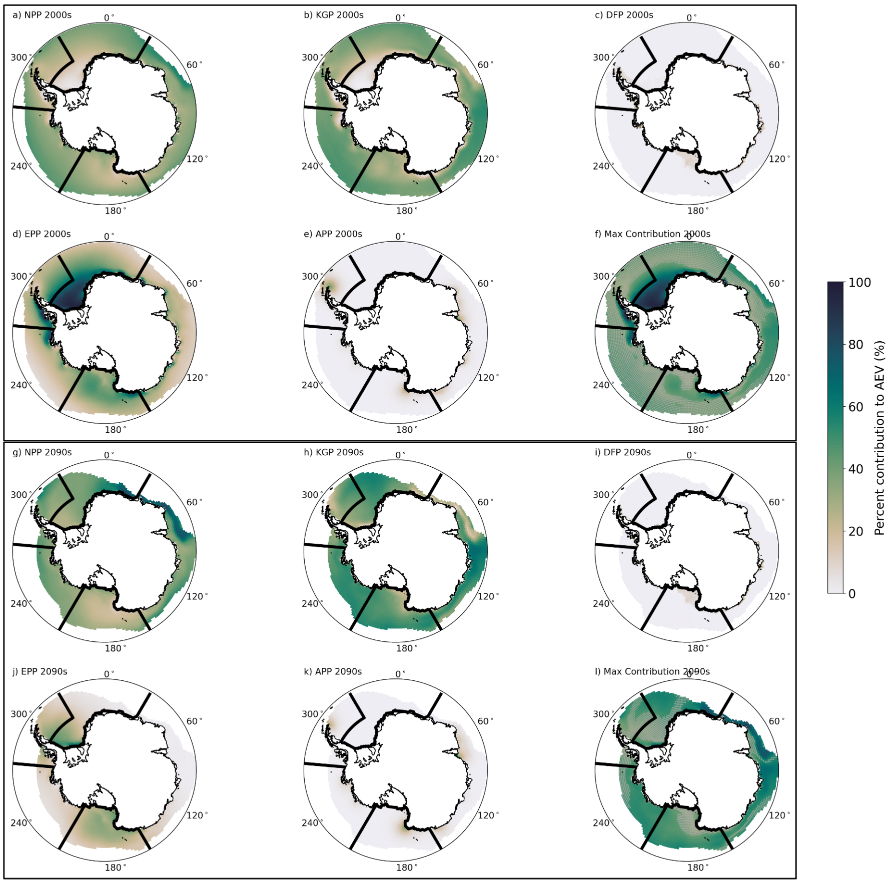

Supplementary Figure 4 Modeled Antarctic Ecosystem Value (AEV) Index input contributions for the 2000s (top box; a-f) and 2090s (bottom box; g-l). The percent contribution at each grid point is provided for net primary productivity (NPP; a and g), krill growth potential (KGP; b and h), demersal fish biomass potential (DFP; c and i), Emperor penguin (*Aptenodytes forsteri*) population (EPP; d and j), and e) Adélie penguin (*Pygoscelis adeliae*) population (APP; e and k). Values range from 0-100 and sum to 100 over all input layers. The maximum input contribution at each point is shown in panels f) and l) where grey stippling indicates points where the maximum input contribution is less than 10% different from the next highest input layer percent contribution. Coastlines were provided by the US National Ice Center [10].

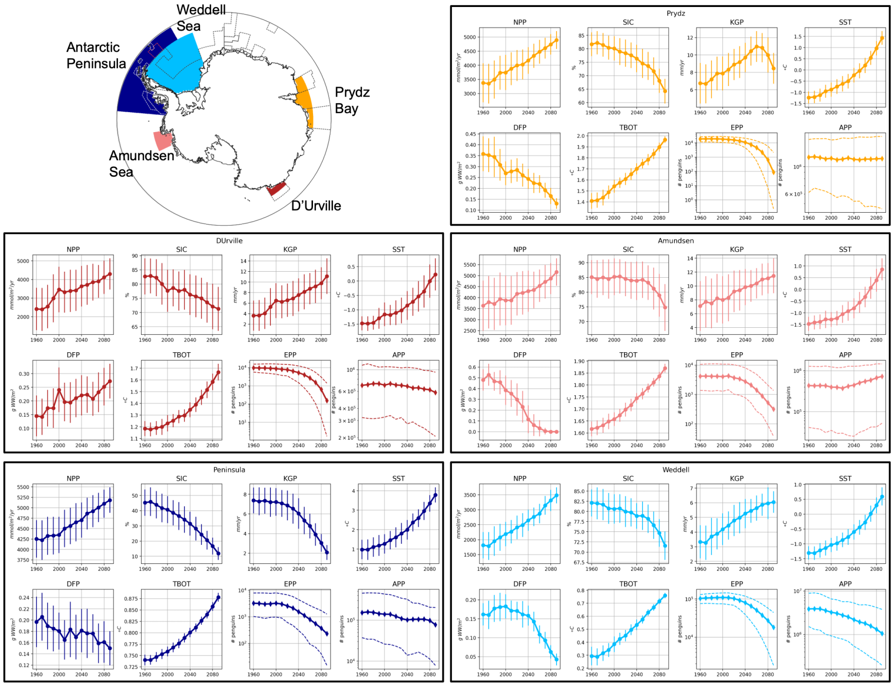

Supplementary Figure 5 Regional biological and environmental variables for Prydz Bay (orange), D'Urville Station (dark red), Amundsen Sea (pink), Antarctic Peninsula (dark blue), and Weddell Sea (light blue) regions as shown on the map with adopted or proposed Marine Protected Areas (MPAs) in dashed lines. Plots for each region are shown below in the black boxes with the same variables and layout but colors matching the regional shading. Variables shown are: (top row from left to right) summer (months ONDJFM) net primary productivity (NPP), spring (months OND) sea ice concentration (SIC), summer (months ONDJFM) krill growth potential (KGP), end of summer (March) sea surface temperature (SST), and (bottom row from left to right) annual mean demersal fish biomass potential (DFP), annual mean bottom temperature (TBOT), Emperor penguin total population (EPP), Adélie penguin total population (APP). All plots, except EPP and APP, are area weighted averages over the shaded region and standard deviation is shown with error bars; each of these decadal statistics was calculated from 250 samples (50 ensemble members * 5 years surrounding the decade). EPP and APP are the total population over the relevant region where the solid line is the median projection and the dashed lines are the 95th percent confidence intervals directly from the metapopulation model projections. All panels have linear y axes except for EPP and APP, which have a logarithmic y axis. Coastlines were provided by the US National Ice Center [10]; adopted MPA boundaries were provided by Commission for the Conservation of Antarctic Marine Living Resources (CCAMLR) [11] and proposed MPA boundaries were provided by the Australian Antarctic Division (East Antarctic), Instituto Antrtico Argentino (Domain 1), Alfred Wegner Institute (Domain 3), and Norwegian Polar Institute (Domain 4).

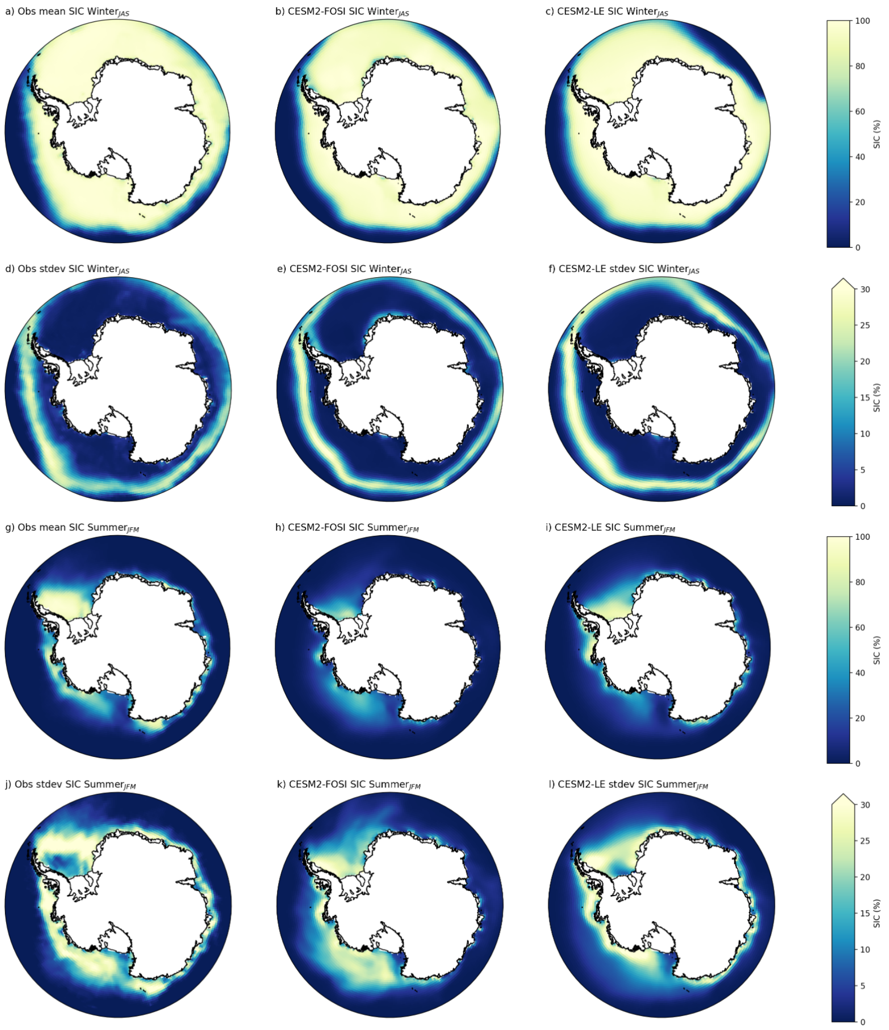

Supplementary Figure 6 Community Earth System Model version 2 (CESM2) sea ice concentration (SIC) evaluation with respect to observations. Both the Mean SICs are shown for winter (a-c, months JAS) and summer (g-i, months JFM); standard deviation for winter (d-f) and summer (j-l) are also shown. Sea ice observations are the Climate Data Records (CDR) of gridded SIC data from satellite images, developed at the National Snow & Ice Data Center (NSIDC) for the National Oceanic and Atmospheric Administration [NOAA; 12, 13]. Data from both the CESM2 forced ocean-sea ice model (CESM2-FOSI) and CESM2 Large Ensemble (CESM2-LE) ensemble mean are shown, and means for the model and observations are taken over the 1979 to 2020 time period. Coastlines were provided by the US National Ice Center [10].

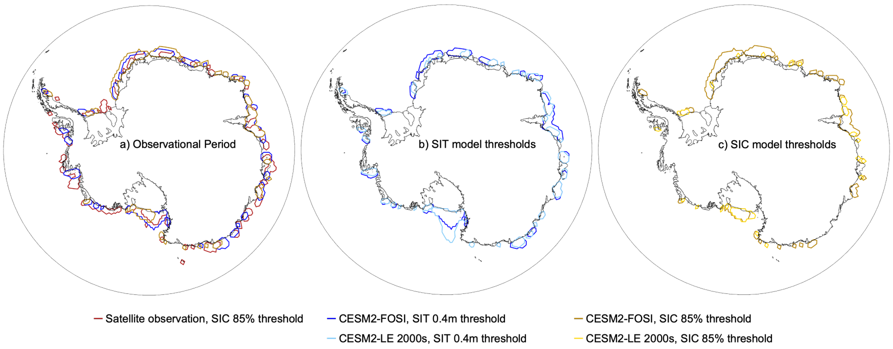

Supplementary Figure 7 Typical present-day polynya areas identified a) over the observational data from satellite observations (red) using a sea-ice concentration (SIC) metric and the Community Earth System Model version 2 (CESM2) forced ocean-sea ice model (CESM2-FOSI) experiment using a sea-ice thickness metric (dark blue) and a sea-ice concentration metric (dark gold). Panel b) compares the sea-ice thickness (SIT) metric polynya areas from the CESM2-FOSI experiment (dark blue, same as in panel a) with those identified from the CESM2 Large Ensemble (CESM2-LE; light blue). Panel c) compares the sea-ice concentration (SIC) metric polynya areas from the CESM2-FOSI experiment (dark gold, same as in panel a) with those identified from the CESM2-LE (light gold). Means for the model and observations are taken over the 1979 to 2020 time period. Coastlines were provided by the US National Ice Center [10].

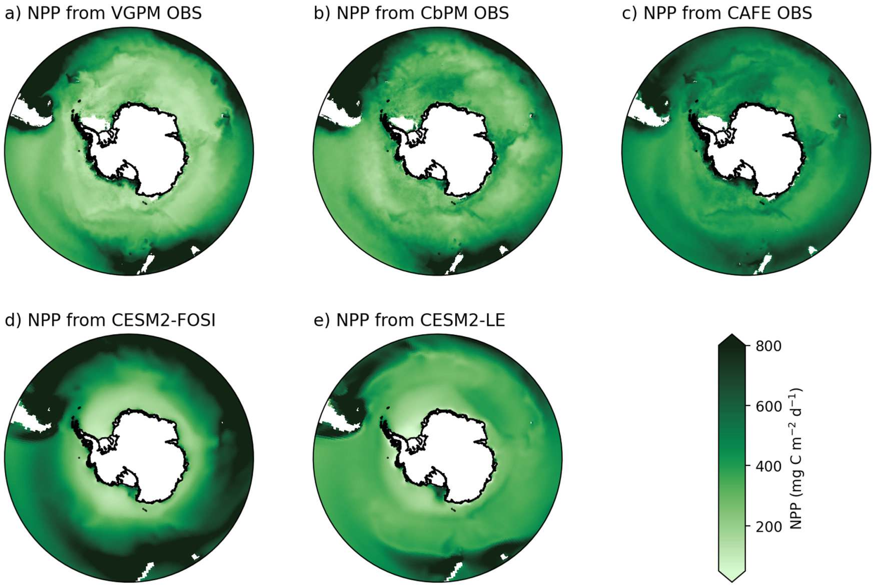

Supplementary Figure 8 Net primary productivity (NPP) over the Antarctic growing season (October-March) for a) MODIS satellite-based vertically generalized production model [VGPM; 14], b) MODIS satellite-based carbon-based productivity model [CbPM; 15], c) MODIS satellite-based carbon, absorption, and fluorescence euphotic-resolving NPP model [CAFE; 16], d) Community Earth System Model version 2 (CESM2) forced ocean-sea ice model (CESM2-FOSI), and e) CESM2 Large Ensemble (CESM2-LE) ensemble mean. Means for both the model and observations are taken over the 2003 to 2018 time period. Coastlines were provided by the US National Ice Center [10].

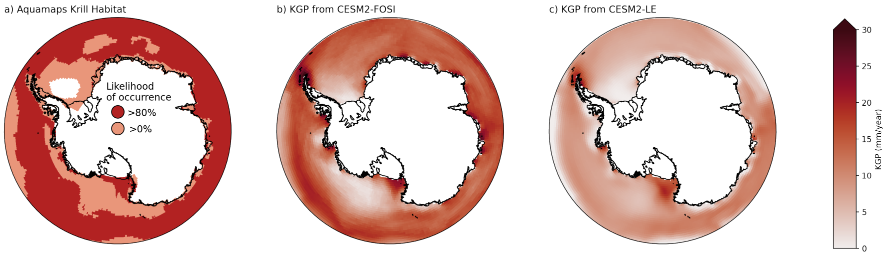

Supplementary Figure 9 Antarctic krill (*Euphausia superba*) a) habitat estimate for > 80% and > 0% likelihood of occurrence from Aquamaps [17] and growth potential model [3] using b) the Community Earth System Model version 2 (CESM2) forced ocean-sea ice model (CESM2-FOSI), and c) CESM2 Large Ensemble (CESM2-LE) ensemble mean. Means for the model are taken over the 1979 to 2020 time period. Coastlines were provided by the US National Ice Center [10].

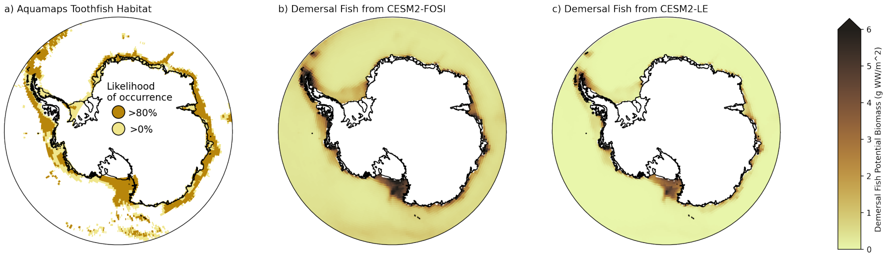

Supplementary Figure 10 Antarctic toothfish (*Dissostichus mawsoni*) a) habitat estimate for > 80% and > 0% likelihood of occurrence from Aquamaps [17] and demersal fish biomass potential from the Fisheries Size and Functional Type (FEISTY) model [4], run using b) the Community Earth System Model version 2 (CESM2) forced ocean-sea ice model (CESM2-FOSI), and c) CESM2 Large Ensemble (CESM2-LE) ensemble mean. Means for the model are taken over the 1979 to 2020 time period. Coastlines were provided by the US National Ice Center [10].

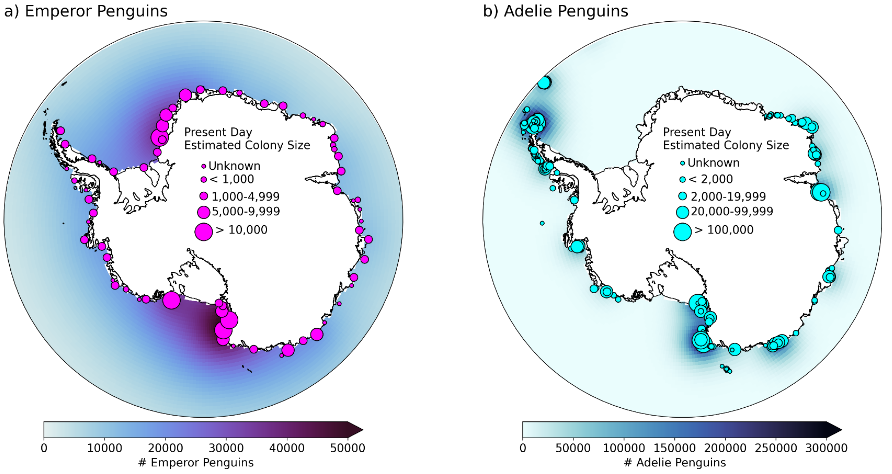

Supplementary Figure 11 Satellite based present day colony population estimates and estimated accessibility of ocean grid points for a) Emperor penguins (*Aptenodytes forsteri*) with satellite estimates from 5 and b) Adélie penguins (*Pygoscelis adeliae*) with satellite estimates from 7. Coastlines were provided by the US National Ice Center [10].

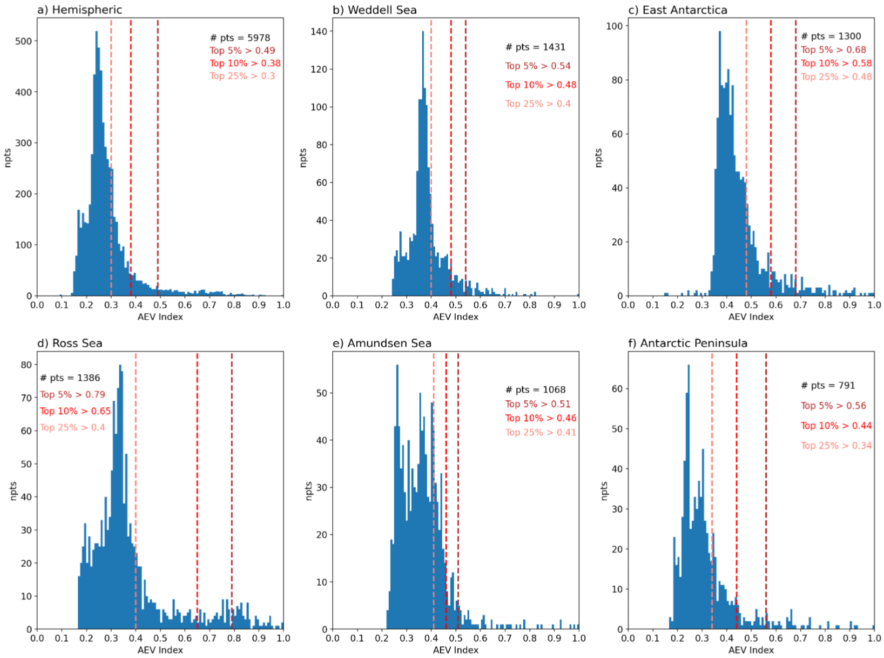

Supplementary Figure 12 Distribution of Antarctic Ecosystem Value (AEV) Index data for the historical reconstruction for a) hemispheric, b) Weddell Sea, c) East Antarctic, d) Ross Sea, e) Amundsen Sea, and f) Antarctic Peninsula regions. Dashed lines and text show the exceptional (top 5th percentile value; dark red), very high (top 10th percentile value; red), and high (top 25th percentile value; pink) AEV Index thresholds. In all panels, the y-axis is the number of grid points (npts). Regions correspond to those shown on Figure 1a,b in Main Text, and the points that correspond to the exceptional, very high, high, and other bins are shown on Figure 1c,d in Main Text.

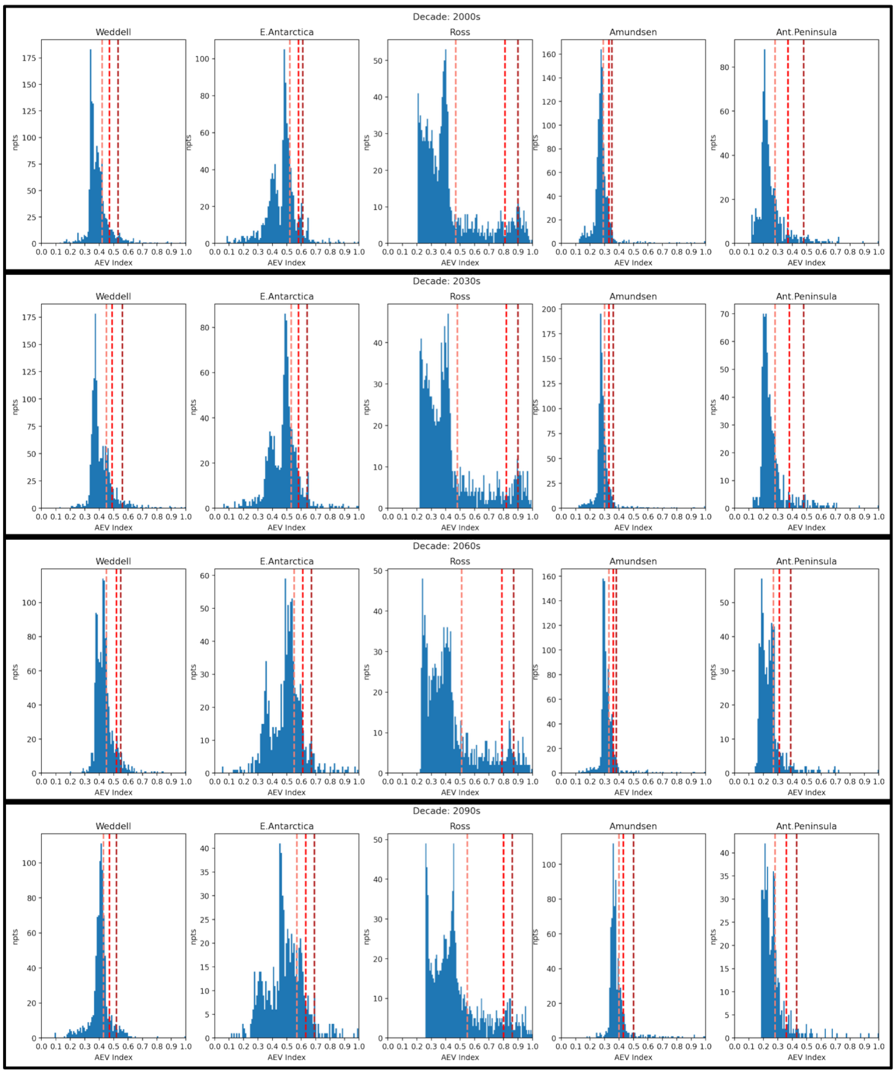

Supplementary Figure 13 Distribution of Antarctic Ecosystem Value (AEV) Index values for the Earth system model data for the 2000s (top row), 2030s (2nd row), 2060s (3rd row), and 2090s (bottom row). Regions shown are the Weddell Sea (left column), East Antarctic (2nd column), Ross Sea (3rd column), Amundsen Sea (4th column), and Antarctic Peninsula (right column). Dashed lines show the exceptional (top 5th percentile value; dark red), very high (top 10th percentile value; red), and high (top 25th percentile value; pink) AEV Index thresholds for each decade and region. In all panels, the y-axis is the number of grid points (npts). Regions correspond to those shown on Figure Figure 1b in Main Text, and the points that correspond to the exceptional, very high, high, and other bins are shown on Figure 4a-d in Main Text.

3 Supplementary References

References

[1] Krumhardt, K.M., Long, M.C., Petrik, C.M., Levy, M., Castruccio, F.S., Lindsay, K., Romashkov, L., Deppenmeier, A.-L., Denchre, R., Chen, Z., Landrum, L., Danabasoglu, G., Chang, P.: From nutrients to fish: Impacts of mesoscale processes in a global CESM-FEISTY eddying ocean model framework. Progress in Oceanography **227**, 103314 (2024) https://doi.org/10.1016/j.pocean.2024.103314

[2] Rodgers, K.B., Lee, S.-S., Rosenbloom, N., Timmermann, A., Danabasoglu, G., Deser, C., Edwards, J., Kim, J.-E., Simpson, I.R., Stein, K., Stuecker, M.F., Yamaguchi, R., Bdai, T., Chung, E.-S., Huang, L., Kim, W.M., Lamarque, J.-F., Lombardozzi, D.L., Wieder, W.R., Yeager, S.G.: Ubiquity of human-induced changes in climate variability. Earth System Dynamics **12**(4), 1393–1411 (2021) https://doi.org/10.5194/esd-12-1393-2021

[3] Atkinson, A., Shreeve, R.S., Hirst, A.G., Rothery, P., Tarling, G.A., Pond, D.W., Korb, R.E., Murphy, E.J., Watkins, J.L.: Natural growth rates in Antarctic krill (Euphausia superba): II. Predictive models based on food, temperature, body length, sex, and maturity stage. Limnology and Oceanography **51**(2), 973–987 (2006) https://doi.org/10.4319/lo.2006.51.2.0973

[4] Petrik, C.M., Stock, C.A., Andersen, K.H., van Denderen, P.D., Watson, J.R.: Bottom-up drivers of global patterns of demersal, forage, and pelagic fishes. Progress in Oceanography **176**, 102124 (2019) https://doi.org/10.1016/j.pocean.2019.102124

[5] LaRue, M., Iles, D., Labrousse, S., Fretwell, P., Ortega, D., Devane, E., Horstmann, I., Viollat, L., Foster-Dyer, R., Le Bohec, C., Zitterbart, D., Houstin, A., Richter, S., Winterl, A., Wienecke, B., Salas, L., Nixon, M., Barbraud, C., Kooyman, G., Ponganis, P., Ainley, D., Trathan, P., Jenouvrier, S.: Advances in remote sensing of emperor penguins: first multi-year time series documenting trends in the global population. Proceedings of the Royal Society B: Biological Sciences **291**(2018), 20232067 (2024) https://doi.org/10.1098/rspb.2023.2067

[6] Jenouvrier, S., Holland, M., Stroeve, J., Serreze, M., Barbraud, C., Weimerskirch, H., Caswell, H.: Projected continent-wide declines of the emperor penguin under climate change. Nature Climate Change **4**, 4 (2014) https://doi.org/10.1038/nclimate2280

[7] Lynch, H.J., LaRue, M.A.: First global census of the Adlie Penguin. The Auk **131**(4), 457–466 (2014) https://doi.org/10.1642/AUK-14-31.1

[8] Jenouvrier, S., Garnier, J., Patout, F., Desvillettes, L.: Influence of dispersal processes on the global dynamics of Emperor penguin, a species threatened by climate change. Biological Conservation **212**, 63–73 (2017) https://doi.org/10.

1016/j.biocon.2017.05.017

[9] Hindell, M.A., Reisinger, R.R., Ropert-Coudert, Y., Hckstdt, L.A., Trathan, P.N., Bornemann, H., Charrassin, J.-B., Chown, S.L., Costa, D.P., Danis, B., Lea, M.-A., Thompson, D., Torres, L.G., Van De Putte, A.P., Alderman, R., Andrews-Goff, V., Arthur, B., Ballard, G., Bengtson, J., Bester, M.N., Blix, A.S., Boehme, L., Bost, C.-A., Boveng, P., Cleeland, J., Constantine, R., Corney, S., Crawford, R.J.M., Dalla Rosa, L., De Bruyn, P.J.N., Delord, K., Descamps, S., Double, M., Emmerson, L., Fedak, M., Friedlaender, A., Gales, N., Goebel, M.E., Goetz, K.T., Guinet, C., Goldsworthy, S.D., Harcourt, R., Hinke, J.T., Jerosch, K., Kato, A., Kerry, K.R., Kirkwood, R., Kooyman, G.L., Kovacs, K.M., Lawton, K., Lowther, A.D., Lydersen, C., Lyver, P.O., Makhado, A.B., Mrquez, M.E.I., McDonald, B.I., McMahon, C.R., Muelbert, M., Nachtsheim, D., Nicholls, K.W., Nordy, E.S., Olmastroni, S., Phillips, R.A., Pistorius, P., Pltz, J., Ptz, K., Ratcliffe, N., Ryan, P.G., Santos, M., Southwell, C., Staniland, I., Takahashi, A., Tarroux, A., Trivelpiece, W., Wakefield, E., Weimerskirch, H., Wienecke, B., Xavier, J.C., Wotherspoon, S., Jonsen, I.D., Raymond, B.: Tracking of marine predators to protect Southern Ocean ecosystems. Nature **580**(7801), 87–92 (2020) https://doi.org/10.1038/s41586-020-2126-y

[10] Gerrish, L., Fretwell, P., Cooper, P.: Medium resolution vector polygons of the Antarctic coastline (7.3). UK Polar Data Centre, Natural Environment Research Council, UK Research & Innovation (2020). https://doi.org/10.5285/ed0a7b70-5adc-4c1e-8d8a-0bb5ee659d18 . https://usicecenter.gov/Resources/AntarcticShelf

[11] Commission for the Conservation of Antarctic Marine Living Resources (CCAMLR): CCAMLR Geographic Information System (GIS). https://gis.ccamlr.org/ Accessed 2024-11-14

[12] Meier, W.N., Peng, G., Scott, D.J., Savoie, M.H.: Verification of a new NOAA/NSIDC passive microwave sea-ice concentration climate record. Polar Research **33**(0), 21004 (2014) https://doi.org/10.3402/polar.v33.21004

[13] Meier, W., Fetterer, F., Windnagel, A.K., Stewart, J.S.: NOAA/NSIDC Climate Data Record of Passive Microwave Sea Ice Concentration. National Snow and Ice Data Center, Boulder, Colorado, USA (2021). https://doi.org/10.7265/efmz-2t65 . https://nsidc.org/data/g02202/versions/4 Accessed 2023-09-30

[14] Behrenfeld, M.J., Falkowski, P.G.: Photosynthetic rates derived from satellite-based chlorophyll concentration. Limnology and Oceanography **42**(1), 1–20 (1997) https://doi.org/10.4319/lo.1997.42.1.0001

[15] Westberry, T., Behrenfeld, M.J., Siegel, D.A., Boss, E.: Carbon-based primary productivity modeling with vertically resolved photoacclimation. Global Biogeochemical Cycles **22**(2) (2008) https://doi.org/10.1029/2007GB003078

[16] Silsbe, G.M., Behrenfeld, M.J., Halsey, K.H., Milligan, A.J., Westberry, T.K.: The cafe model: A net production model for global ocean phytoplankton. Global Biogeochemical Cycles **30**(12), 1756–1777 (2016) https://doi.org/10.1002/2016GB005521

[17] Kaschner, K., Kesner-Reyes, K., Garilao, C., Segschneider, J., Rius-Barile, J., Rees, T., Froese, R.: AquaMaps: Predicted range maps for aquatic species (2019). https://www.aquamaps.org
